# Separate collection and bio-waste valorization in the Italian poultry sector by material flow analysis

**DOI:** 10.1007/s10163-022-01366-0

**Published:** 2022-02-09

**Authors:** Christian Bux, Vera Amicarelli

**Affiliations:** Department of Economics, Management and Business Law, University of Bari Aldo Moro, Bari, Italy

**Keywords:** Poultry industry, Material flow analysis, Sustainable consumption, Food waste, Separate collection

## Abstract

Poultry meat production and consumption face several challenges under economic, social and environmental perspectives, and increasing concerns are associated with food loss and waste minimization. One of the main issues is related to the absence of a homogeneous and standardized separate collection of bio-waste at country level, which makes chicken bones, skin and food waste valorization a challenging goal. The present research, implementing the material flow analysis to the Italian poultry sector, aims at measuring poultry-related co-products and by-products, exploring food waste, chicken bones and skin and the current trends in the Italian bio-waste separate collection. Then, it discusses alternative separate collection strategies and sustainable consumption habits. Data have been collected according to a research triangulation approach, whereas system boundaries consider slaughterhouse, distribution and final consumption stage. It emerges that more than 1.50 Mt of live animals have been processed to obtain 0.46 Mt of fresh meat and 0.76 Mt of co-products and by-products, of which more than 0.32 Mt are represented by chicken bones and skin. In addition, more than 0.15 Mt of food waste have been recorded. The research adds an extra step towards the identification of awareness campaigns and separate collection strategies at national level.

## Introduction

Poultry meat production and consumption face several challenges under economic, social and environmental perspectives, and increasing concerns are associated with food loss and waste minimization. At present, European poultry meat production has increased (and still increases) at high rates, reaching an upsurge by 20% from 2014 to 2019 [1]. Europe represents the third market player in the poultry sector, being Poland the first producer (16%), followed by Turkey (13%), Spain and France (10%) [2]. In terms of companies, the largest European ones are localized in France, Netherlands and Italy, with, respectively, 541, 426 and 350 million animals slaughtered in 2019 [3].

The United Nations, through the proposal of the Sustainable Development Goals (SDGs), have addressed several strategies towards climate change reduction and human well-being in the field of agri-food industries [4]. Livestock production, meat processing and consumption, as well as food waste generation, impose concrete challenges either under social, environmental or financial perspective, and high rate of natural resource consumption in terms of water and energy. Therefore, environmental inventories and circular strategies become crucial [5]. It is estimated that the entire agri-food sector, from agricultural production to household and food service consumption, is responsible for approximately 29% of the entire humanity’s ecological footprint [6], highlighting the urgency to assess food production and consumption related-hidden costs [7, 8]. The agribusiness, defined as the “sum of all operations involved in manufacture and distribution of farm supplies, production operations on the farm, and the storage, processing, and distribution of farm commodities”, must be able to maximize its profit without compromising consumers satisfaction and environmental needs [9, 10]. Currently, it represents one of the major land users, as well as one of the main responsible of biodiversity and ecosystems alteration, causing more than 80% of global deforestation, 70% of freshwater use and terrestrial biodiversity loss, 52% of degraded agricultural land, 50% of freshwater biodiversity loss and 29% global greenhouse gases emissions [11–13]. In addition, the recent national and international strategies (e.g., “SDGs”, “Closing the Loop—An EU Action Plan for Circular Economy”, “Farm to Fork strategy”) have estimated food waste issue in more than 1.3 billion tons/per year of food thrown away along the entire supply system. This amount represents approximately 3.3 Gigatons (Gt) of CO_2_ equivalent (6% of total greenhouse gases emission), more than USD 1 trillion per year, over 250 km^3^ of blue water footprint and approximately 1.4 billion hectares of land losses [14–16].

The present research, through the application of the material flow analysis (MFA), explores the Italian poultry production, highlighting the importance of co-products and by-products sorting and valorization towards agribusiness sustainability and circularity. At present, the Italian meat industry is subject to the Regulation (EC) 1069/2009, which addresses the agri-food sector circularity and forces, among others, food waste minimization and proper use, assuring public and animal health, food safety, environmental sustainability and consumers’ confidence [17]. In addition, being food waste disposal a “not realistic option”, the legislative framework highlights the urgency for food waste measurement (i.e., quantification and qualification), collection and valorization, either guaranteeing financial or environmental benefits.

In the light of these premises, the present research aims at measuring poultry-related co-products and by-products, estimating food waste, bones and skin quantities from slaughterhouse to final consumption and analyzing the current trends in the Italian municipal separate collection. Further, considering the absence of a homogeneous and standardized separate collection of bio-waste at country level, it discusses alternative separate collection strategies and sustainable consumption habits towards bio-waste reduction. Although several studies applied the MFA in the agri-food sector [18, 19] with reference to food waste [20, 21], and several researches evaluated bones valorization opportunities [22–24], none of them assesses their quantity and evaluates their quality in the Italian poultry meat system, helping academics, managers or public authorities to enhance national separate collection targets and reach SDGs proposals. The present research tries to add an extra step in bio-waste sorting and valorization, highlighting the need to adopt homogeneous and standardized separate collection techniques.

### Literature review and current trends in poultry production and consumption

The global meat consumption is expected to increase in the next decade on a global scale, either in developed or developing countries such as Asia, Latin America, Central and Eastern Europe [5]. As reported by official statistics [25], the leading country in terms of consumption is North America (over 95 kg per capita per year), followed by Oceania (70 kg) and Europe (65 kg). In addition, the average meat consumption is expected to increase at a constant rate, from 34 kg per capita in 2018 to roughly 35 kg in 2028. Among European countries, the average consumption, affected by economic development, well-being and culture differences [26], is nearly twice the global average. In terms of meat type, poultry meat represents the second most consumed typology (24 kg per capita per year) after pork (32.5 kg), with positive consumption outlooks.

As far as the production side, Italy represents an interesting case in European reality, slaughtering more than 600,000 heads in 2020 [2] and recording an increase by 13.4% compared to 2010. Moreover, the global number of Italian animals has been bred within approximately 2,690 farms essentially localized in Northern Italy (i.e., Veneto, Emilia-Romagna and Lombardy), more than 90% of national slaughtered animals have been processed by two single companies (i.e., Gruppo Veronesi, Amadori) and the total revenues has been estimated in more than USD 11 billion dollars in 2020, with an expected increase equal to USD 12 billion in 2024 [27]. In addition, considering the average European rate, the Italian poultry sector represents a concrete example of self-sufficiency intending its ability to cover all domestic consumption by domestic production. The poultry “net production” is equal to the “internal production” and records a self-sufficiency rate of more than 108%, which is expected to increase in the next few years [28, 29]. The increase in domestic production favors esports, stimulating an enhancement in the Italian trade balance. Further, an increase in internal production provokes an increase in individuals’ consumption. Related to this last issue, an exceptional surge in poultry sales compared to other meat typologies [29] has been recorded because of the COVID-19 pandemic effects that have imposed unpredictable lifestyles, improvements in smart food delivery and never experienced time management. Poultry industry shows a faster expansion to respond to an increasing demand for protein and low-fat meat but consequently its waste production grows equally fast, mainly composed of food waste, emitting over 0.6 Gt of CO_2_ equivalent (18% of global food waste-related emissions) [30, 31]. To improve companies’ environmental performances and enhance environmental entrepreneurship, strong waste management policies at local level are required. On the one hand, sustainable waste management practices should encourage landfill diversion through recycling, composting or energy recovery [32], enabling municipalities and single companies to produce high-added value products or biofuels (e.g., biodiesel, bio-methane) [33]. On the other hand, academics have highlighted the need to enhance waste management planning by combining material and substance flow analyses in a holistic and integrated approach [34], at the same time maximizing environmental savings and minimizing management and disposal costs [35]. Several authors have analyzed poultry production environmental impacts, from agricultural stage [36, 37] to retail, highlighting the significant role of feed production, farm management and transportation towards climate change, but final consumption research is still missing. In addition, although some previous research has investigated the poultry system in Southern Italy [38] and more recent authors have investigated local experiences in Central Italy [39], Italian poultry industry-related co-products, by-products or food waste potential seems to be still unexplored.

## Research methodology

### Material flow analysis and food waste definition

In line with the Commission Delegated Decision (UE) 2019/1597 [40], the research applies the MFA to assess, under quantitative and qualitative perspective, Italian poultry system-related co-products, by products and food waste, investigating the amount of food waste, chicken bones and skin on a circular economy basis. The Delegated Decision sets a “common methodology and minimum quality requirements for the uniform measurement of levels of food waste”, imposing on European Member States to report by 30 June 2022 on food waste generated in 2020 through a quality check report. In addition, such a decision states that food waste amounts should be measured and reported for each stage of the supply chain at least once every 4 years [41]. Although several food waste definitions have been proposed worldwide, an amendment to Directive 2008/98/EC [42] defines food waste as “all food that has become waste”, including among them also inedible parts not separated from the edible parts such as bones attached to meat destined for human consumption (i.e., parts of food intended to be ingested or not). Therefore, food waste occurs along the entire food supply chain, either at upstream, core or downstream stages. The present research distinguishes between edible and inedible parts, intending as food waste those edible parts not addressed to human consumption, and defining inedible parts as co-products, by-products, bones and skin. Both edible and inedible parts are defined as bio-waste.

The MFA, defined as a “systematic assessment of the state and change of materials flow and stock in space and time” [43], has been successfully applied in literature, demonstrating its utility in evaluating single products, industrial sectors or entire countries socio-economic metabolism [44–46]. In addition, the MFA could: (i) provide an estimation of food waste currently generates at European Member States level; (ii) compare and complement the amounts reporter by each Member State, representing a possible benchmark; and (iii) provide a consistent overview of food waste evaluation, informing on possible reduction targets [47].

**Fig. 1:**
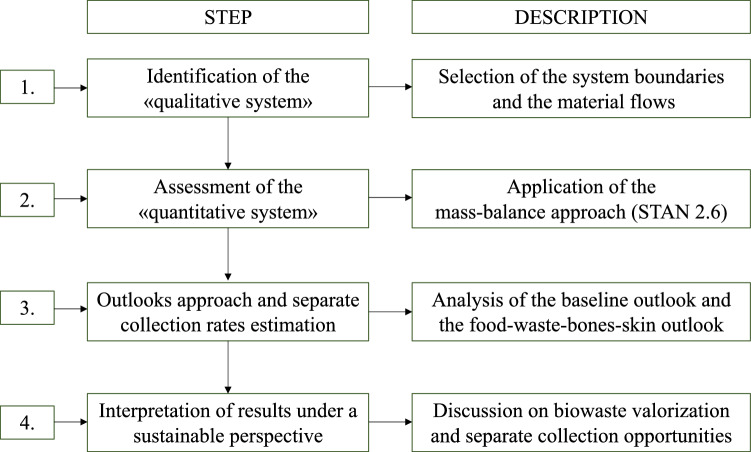
Research algorithm and stepwise approach. Source: Personal elaboration by the authors

The present research has applied a stepwise approach [48] and Fig. 1 illustrates the research algorithm, distinguishing among four different steps, as follows: (i) definition of material flows and identification of the “qualitative” system; (ii) calculation of flows (“quantitative” system); (iii) outlooks approach and separate collection rates estimation; (iv) interpretation of results under a sustainable perspective. As regards the bio-waste separate collection rates in Italy, Ispra [49] has been considered. Data have been processed through STAN 2.6. (substance flow ANalysis), developed by the Institute for Water Quality, Resources and Waste Management at Vienna University of Technology to balance material and substance flows within a specific system.

**Fig. 2:**
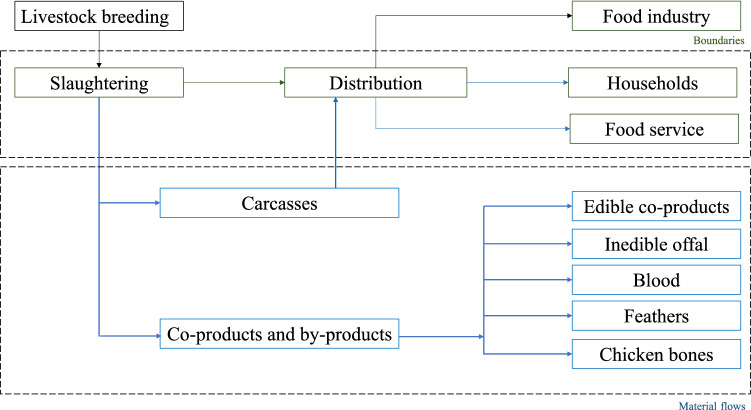
Description of the “poultry meat system”. Green lines indicate system boundaries, blue lines indicate material flows. Source: Personal elaboration by the authors

In terms of system boundaries and material flows (Fig. 2), the research investigates fresh poultry meat production from slaughterhouse to final consumption, either considering food service or household consumption. Upstream stages (i.e., farm management, feed storage, livestock breeding) and related food waste (e.g., dead animals) are out of boundaries, as well as the food processing industry (e.g., chicken meat-based products, nuggets). Furthermore, because the Italian poultry system could be considered 100% self-sufficient [1, 29], a closed economy scenario has been investigated, not accounting for either import or export.

### Data collection

To perform an analytical mass balance research, the authors have adopted both top-down and bottom-up approaches, including either official statistics [50], scientific articles or national and international reports [1, 29, 51–53]. First, data are essentially related to chickens and hens (synonym for poultry, from now on) representing on national scale over 94% of total slaughtered heads, while other meat subcategories, such as turkeys (4.8%), guinea fowl (0.7%), geese (0.1%), have not been accounted. Table 1 shows national data, providing the number of slaughtered animals, total live weight, average live weight per animal, total dead weight and national incidence (%) in 2020.

**Table 1:** Slaughtering in Italian poultry industry in 2020 considering: number of heads of slaughtered animals; total live weight expressed in tons; average live weight per head expressed in kg, total dead weight expressed in tons and national incidence, intending the percentage of animals born and bred in Italy out of the total

Poultry type	Slaughtered animals (thousands)	Total live weight (t)	Average live weight per head (kg)	Total dead weight (t)	National incidence (%)
Chickens	573,845,774	1,505,625	2.6	1,066,943	93.9
Turkeys	29,431,211	417,261	14.2	313,248	4.8
Guinea fowls	4,198,229	7,703	1.8	5,449	0.7
Geese	714,104	2,237	3.1	1,697	0.1
Game	3,202,093	3,202	0.2	2,16	0.5
Total	611,391,411	430,403	–	322,554	100

Source: Personal elaboration by the authors on Istat [50]

The poultry yield (dead weight/live weight) is estimated at 70.86%. As far as concerns carcass allocation at slaughterhouse gate, as well as fresh meat, co-products and by-products flows along the entire food supply chain, it has been estimated that more than 75% of carcasses are destined to retail, 12% to food service (i.e., intending canteens, restaurants, hotels) and 13% to food industry. This last percentage is out-of-boundaries and refers to the production of chicken meat-based products such as nuggets, patties, burgers, sausages, and others. At the retail stage, more than 40–45% of fresh meat is distributed within modern retail shops (i.e., large-scale distribution), while approximately 55–60% at traditional retail (i.e., butchers). The poultry industry shows a significant difference compared to other meat industries (e.g., pork and beef) where an additional deboning occurs at retail and slaughterhouse, considering that whole chickens, as well as chicken wings and thighs, are usually sold and consumed at households with bones (so-called “meat on the bone” or “bone-in meat”).

### Outlooks, separate collection rates and general assumptions

The authors investigate two different outlooks: (i) the baseline one, which explores material streams without considering chicken bones, skin and food waste weight along the entire food supply chain, from slaughterhouse to final consumption; (ii) the food waste–bones–skin (FWBS) outlook, which includes bones, skin and food waste streams, and focuses on food service and final consumption stages. The examination of different outlooks helps in comparing alternative perspectives and states of a system, accounting for different parameters and forecasting optimistic or pessimistic performances [54, 55]. As regards the second outlook, which includes in material flows calculations also bones, skin and food waste percentages, average component yields of chicken carcass have been considered, as follows: (i) fresh meat (59%); bones (25%); skin (16%) [56, 57]. Food waste percentages at distribution and food service/households have been estimated, respectively, at 12% and 3% [52]. Table 2 illustrates the alternative outlooks percentages.

**Table 2:** Outlooks investigation and bones, skin and food waste percentages

Outlook	Slaughterhouse				Distribution	Retail			Final consumption		
	Co-products	In. offal	Blood	Feathers	FW	FW	Bones	Skin	FW	Bones	Skin
Baseline	2%	18%	4%	6%	N/A	N/A	N/A	N/A	N/A	N/A	N/A
FWBS	2%	18%	4%	6%	12%	3%	25%	15%	3%	25%	15%

Source: Personal elaboration by the authors

*In.* inedible, *FW* food waste

To fill in data gaps, the authors contacted an Italian company, which is one of the highly involved representatives in the entire beef supply chain, and used the research triangulation to improve the overall validity and credibility of the data. Merging primary and secondary data, the research triangulation allows to exploit the synergistic effects of joining investigative techniques to decrease the bias [58–60]. In addition, considering the majority of data deriving from national reports [1, 29], international documents [2] and national official statistics [50], whereas a few information coming from scientific articles, the present research did not carry out an uncertainty analysis. As already discussed by previous studies applying the mass balance approach [61, 62], the uncertainty assessment would not have added additional value to the research.

## Results

### Material flow analysis (baseline outlook)

The process “slaughtering” within the “poultry meat system” encompassed three main phases [63], as follows: (i) pre-stunning, which includes arrival unloading of containers from the truck, lairage, handling/removing of birds from containers; (ii) stunning, which includes restrain; and (iii) bleeding, which includes bleeding following stunning and bleeding during slaughtering without stunning. The number of live animals entering the system is around 573 million, whose live average weight is 2.6 kg, and the total live weight is 1,505,635 t. Considering an average yield of approximately 71%, it is essential to distinguish the material composition of poultry, since live birds enter the system as inputs, but several components leave the system as outputs.

**Fig. 3:**
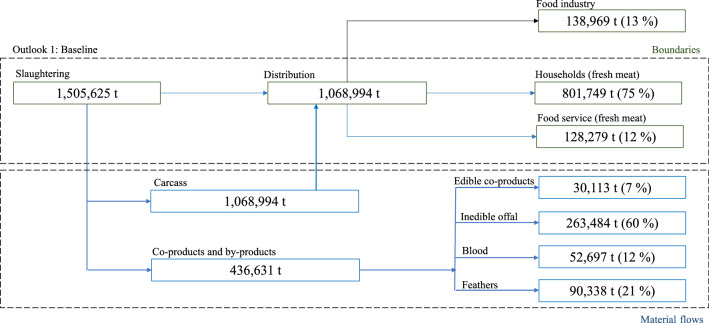
Material flow analysis for the baseline scenario in which chicken bones and skin are not considered within the material flows (in blue). Source: Personal elaboration by the authors

As illustrated by Fig. 3, the material streams flowing within the system are carcass, for an amount of approximately 1,068,994 t and co-products and by-products, distinguished as follows: (i) edible co-products (30,111 t); (ii) inedible offal (263,484 t); (iii) blood (52,697 t); (iv) feathers (90,338 t). Then, carcasses are addressed to three different paths, which are food service (128,179 t), distribution (801,745 t) and food industry (138,969 t). Among “distribution”, it is interesting to distinguish between traditional retail (338,838 t) and modern retail (228,628 t), where a certain amount of material streams is accounted as food waste and/or bones and skin (128,279 t), whereas at food service it is possible to estimate such a variable at 5,773 t. Therefore, the available amount of fresh meat at household consumption amounts to 673,466 t, of which 643,160 t actually for human nutritional intake and approximately 30,306 t represented by food waste, bones and skins.

### Material flow analysis (FWBS outlook)

The first outlook essentially describes the easiest “poultry meat system”, illustrating the poultry flows along the supply chain, from slaughterhouse to consumption. It simply takes care of fresh meat, co-products and by-products, without considering the significant composition of food waste at food service and households, which represents a noteworthy amount on a national scale.

**Fig. 4:**
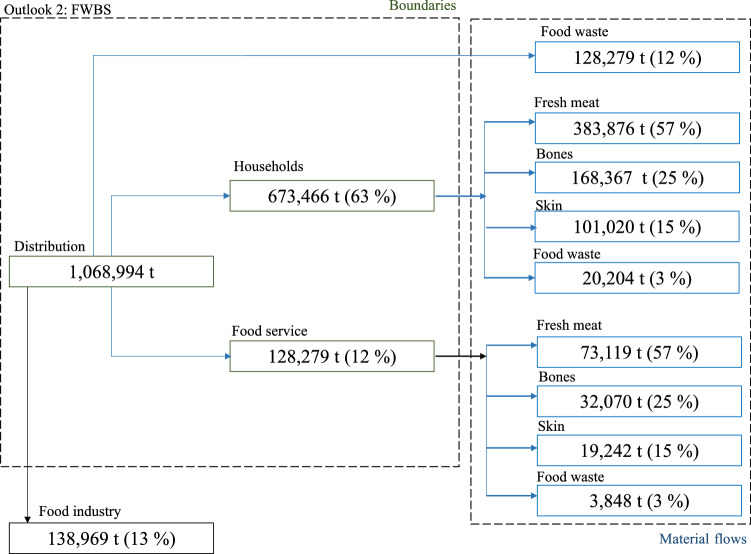
Material flow analysis for the FWBS outlook in which chicken bones and skin are considered within the material flows (blue lines). Source: Personal elaboration by the authors

As illustrated by Fig. 4 (FWBS outlook), which focuses on households and food service and assumes that deboning (i.e., to obtain different meat cuts) occurs at final consumption stage, it is possible to estimate the material flows encompassed within the “poultry meat system”, distinguishing between fresh meat, bones, skin and food waste properly intended (i.e., thrown away fractions of fresh meat). Specifically, material flows at households have been estimated as follows: (i) fresh meat (383,876 t); (ii) bones (168, 367 t); (iii) skin (101, 020 t); and (iv) food waste (20,204 t), whereas at food service: (i) fresh meat (73,119 t); (ii) bones (32,070 t); (iii) skin (19,242 t); and food waste (3,848 t).

As highlighted by the FWBS outlook, the following ratios emerged: (i) bones/fresh meat = 44%; (ii) skin/fresh meat: 26%; (iii) food waste/fresh meat = 5% either for households or food service individually considered. It means that, for each ton of poultry fresh meat produced, 440 kg of bones, 260 kg of skin and 50 kg of food waste are generated, which means approximately 750 kg (three-quarters of the global final product) of material flows which must be addressed, reused or recovered under circular perspective. Table 3 presents all co-products, by-products, bones, skin and food waste to fresh meat ratio, considering as basis the sum of fresh meat as estimated in FWSB outlook (456,995 t).

**Table 3:** Weight of co-products, by-products, bones, skin and food waste and their ratio to fresh meat, from slaughterhouse gate to households and food services’ consumption

Slaughtering gate	Weight (t)	Ratio to fresh meat
Co-products (edible)	30,113	7%
Indible offal (rendering)	263,484	58%
Blood	52,697	12%
Feathers	90,338	20%
**Food service**	**Weight (t)**	**Ratio to fresh meat**
Bones	32,070	7%
Skin	19,242	4%
Food waste	3,848	1%
**Households**	**Weight (t)**	**Ratio to fresh meat**
Bones	168,367	37%
Skin	101,020	22%
Food waste	20,204	4%

Source: Personal elaboration by the authors

The ratio to fresh meat illustrates the significant impact, in terms of weight, of several poultry meat streams within the “poultry meat system”. At slaughterhouse gate, the main secondary output associated with fresh meat production is represented by inedible offal (58%), followed by feathers (20%) and blood (12%). In addition, either at food service or households, the highest amount is represented by bones and skin, and food waste still embodies a suggestive amount.

### Italian bio-waste separate collection per region

Results from the FWBS outlook reveal an amount of 200,437 t of bones, 120,262 t of skin and 24,052 t of food waste at households and food services. Considering the Italian rate of bio-waste separate collection, recorded by Ispra [49] as follows: (i) 67% at Northern Italy; (ii) 54.1% at Central Italy; (iii) 46.1% at Southern Italy, with an average Italian rate at 58.1%, it is possible to estimate the number of bones, skin and food waste properly collected and addressed at regional level at households and food service (Table 4). The expected consumption rate per region has been estimated on the basis of the average population per region, assuming a homogeneous consumption of poultry meat among Italians [64].

**Table 4:** Food waste, bones and skin production per region (t) and collection rates (%) at Italian regional level

Region	FWBS production per region (t)	FWBS collection rate (%)	FWBS collection per region (t)
	a	b	a x b
Veneto	28,084.42	74%	20,726.30
Trentino-South Tyrol	6,150.67	73%	4,459.24
Lombardy	57,820.14	71%	40,878.84
Marche	8,689.07	69%	5,960.70
Emilia-Romagna	25,563.16	67%	17,204.01
Sardinia	9,330.42	67%	6,251.38
Friuli-Venezia Giulia	6,932.01	67%	4,616.72
Umbria	5,037.45	63%	3,193.74
Valle d'Aosta	718.18	62%	447.43
Piedmont	24,843.59	61%	15,229.12
Abruzzo	7,472.29	60%	4,453.49
Tuscany	21,303.38	56%	11,951.20
Campania	33,109.69	53%	17,448.81
Liguria	8,830.57	50%	4,388.79
Lazio	33,565.68	47%	15,876.57
Basilicata	3,187.06	47%	1,507.48
Apulia	22,937.54	45%	10,413.64
Calabria	11,014.14	45%	4,978.39
Molise	1,729.72	38%	664.21
Sicily	28,431.81	30%	8,387.38
*National target*	*344,751.00*	*65%*	*224,088.15*

Source: Personal elaboration by the authors on Ispra [49] and Statista [64]

As results confirm, the expected rate of FWBS production does not coincide with the expected amount of FWBS collection, creating a disparity between production, collection and recycling among Italian regions, and highlighting the need for implementing/improving awareness-raising policies and collection systems at regional level. On average, approximately 58% of FWBS are collected either at food service or households, while more than 144,000 t of FWBS are still addressed to landfill as unsorted waste. Therefore, it emerges that there is still room to improve national (and local) separate collection performances and reach national targets, thereby improving environmental performances through circularization. In addition, only seven regions out of twenty have already reached the national separate collection target (65%), highlighting the need to improve separate collection among all Italian local realities. The regions which record the most critical values (i.e., far from the mean value) result Sicily, Molise, Calabria, Apulia and Basilicata, being at the same time either the major bottlenecks or the main realities on which to intervene. The most relevant circumstance is represented by the fact that this target should have been reached already in 2012.

## Discussion

### Bio-waste separate collection and valorization

The mass balance results, defined under quantitative and qualitative perspective, open several opportunities for material flows evaluation either for environmental and resource management, both at companies and policy-maker level [19]. The achieved results provide a significant background for the choice of best-suited recycling and/or treatment technology of poultry bones, skin and food waste.

Bio-waste valorization opportunities differ from one food industry to another and depend on quantities and types of materials available, patterns of generation, qualitative and quantitative characteristics and variability [65]. However, although usually considered low-valuable materials, food waste could be valorized into valuable compounds through different techniques, as follows: (a) anaerobic digestion to obtain biogas and biofertilizers; (b) microbial fermentation to obtain bioalcohols, biodiesel and biohydrogen; (c) enzymatic hydrolysis to obtain biopolymers, biochemicals and bioplastics; (d) clarification to obtain value added components; (e) carbonization and activation to obtain bio-sorbents; and (f) incineration, gasification and pyrolysis to obtain bioenergy, biochar and bio-oil [66–68]. Though separate collection rates have increased in the last years by a rate of 23.8% from 2014 to 2018, one of the main Italian challenges are related to sorting and separate collection of bio-waste streams. Italian households and food services are still not homogeneously subjected to mandatory separate collection policies throughout the national territory, increasing disparities between the Italian regions. As reported by Ispra [49], several regions have not yet reached the minimum objectives of separate collection imposed at national level, including quite all the Southern regions such as Sicily, Molise, Calabria, Apulia and Basilicata. It means that several Italian municipalities are losing the theoretical potential deriving from bio-waste sorting and recycling.

**Fig. 5:**
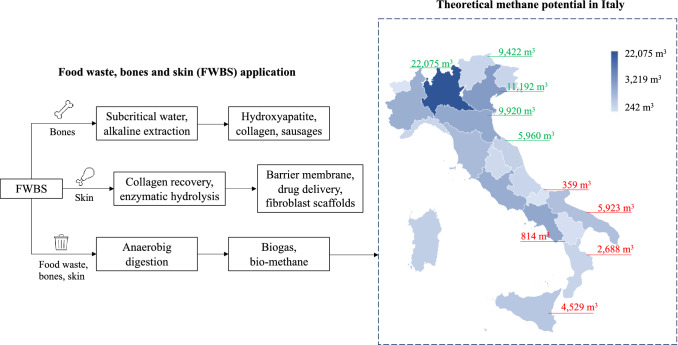
Food waste, bones and skin applications (**a**) and theoretical methane potential in Italy (**b**). Source: Personal elaboration by the authors on Ispra [49] and Statista [64]

Under a circular economy perspective, although several “rethink”, “redesign” or “reuse” rules as proposed by the European Commission Waste Framework Directive cannot be applied in the field of fresh meat production, it is still possible to recycle, compost or recover chicken bones and skin for other purposes (i.e., repurpose) [69]. As stated by Raggi et al. [70], residual flows deriving from one process could be conveniently forwarded to other processes for further uses, recovering them to produce pharmaceutical substances, high-quality commodities, pet food and animal feed, technical materials, fertilizers and biogas. Though the meat sector represents one of the less environmentally friendly industries among food productions, it offers significant opportunities to maximize the conversion of “secondary raw materials” (i.e., food waste, bones, skin) into novel products. In the field of slaughterhouse by-products (e.g., inedible offal, feathers, blood), a variety of applications could be considered [71, 72]. As proposed by Boles et al. [73] and Barakat et al. [74], bones could be converted, adopting the revalorizing technique of the subcritical water or the alkaline extraction, into hydroxyapatite and collagen, as well as in novel kind of meat-based products for human consumption. Then, being skin a significant part of poultry products (16% of its carcass weight), it could be transformed, through techniques like collagen recovery, enzymatic hydrolysis and chromatographic purification, into barrier membrane, drug delivery, fibroblast scaffolds or bioengineered tissues [75, 76]. In addition, considering the average theoretical methane potential (0.52–0.55 m^3^/kg) embedded within food waste as proposed by Wang et al. [77], it is possible to estimate the collected FWBS methane production at approximately 104–110 million m^3^, with the chance of increasing its amount by additional 75–79 million m^3^ if the unsorted FWBS fraction is accounted. Figure 5 summarizes FBWS applications (a) and the theoretical methane potential per region (b).

### Bio-waste reduction and sustainable consumption habits

It is important to highlight that bio-waste valorization opportunities should be based on their *relative value*, intending the number of co-products and by-products concretely reinserted into the anthroposphere. Therefore, it is possible to assume that some municipalities could produce more methane than others but are not exploiting even half of their current potential. Further, several regions have not still reached the national separate collection target, increasing the loss of potential embedded in bio-waste. As highlighted by previous authors [78], the failure in achieving these objectives depends on deficiencies in national waste management systems and illegal traffic activities, as well as on socio-cultural factors, including the lack of involvement of citizens and knowledge on the potential (and impacts) of food waste. Further, the disparities recorded between Northern and Southern regions could be related to the less development managerial culture and the lack of infrastructures. Possible solutions to increase public awareness on the importance of separate collections have been examined by Saladié and Santos-Lacueva [79], who have discussed the importance of television, radio, newspaper or online campaigns. Further, Xu et al. [80] have suggested that economic rewards work better than social influence in enhancing bio-waste separate collection.

On the one hand, technological innovation in the field of Italian separate collection seems to be still limited. On the other hand, innovation in food practices (e.g., sustainable foods, eco consumption habits) could compensate for inefficient municipal waste management by reducing bio-waste production. Considering either the social influence or the concerns about health, animal welfare and environment, an always increasing number of consumers is willing to decrease meat consumption and adopt meat-free diets (e.g., vegans, vegetarians, flexitarians), therefore limiting its related environmental, social and disposal impacts [81]. Several alternative protein sources have emerged in recent years, such as meat substitutes (e.g., grain meat substitutes, seitan, tofu), seaweed, insects and cultured meat [81, 82], which do not account for bones, skin, offal and other co-products and by-products. Italian consumers, according to van de Pas [5], seem to be interested in vegetarians and vegan phenomena since meat-free diets are perceived as healthier, whereas protection of the environment does not represent an important reason to go vegetarian or vegan. Remarkable is the Italian role in the meat substitute market, because it represents the third-largest market, accounting for more than 175 million euros in 2018. On the side of food waste-related social, environmental and financial impacts, the adoption of meat-free diets could represent an interesting opportunity towards sustainability. However, the abandonment of meat consumption does not represent an optimal solution if its related socio-economic consequences are accounted. It is necessary to intervene on individuals’ consumption habits, stressing the role of education towards sustainable behaviors and implementing separate collection at individuals’ level. As outlined by Chriki and Hocquette [82], it is impossible to reproduce the diversity of meats obtained from various species, breeds and cuts, and consumers will always be disappointed by “unnatural food”. Not-consuming does not mean not-wasting: the crucial point is to learn to consume responsibly.

### Separate collection techniques and rates across Europe

Sustainable waste management programs depend on their planning and implementation at local scale. Although a decisive part in the adoption of sustainable sorting and collection behaviors depend on consumers’ attitude towards environmental protection, waste-recycling programs also depend on: (a) raw materials quality; (b) manpower skills; (c) capital funded by local governments and municipalities; (d) technologies available to collect, handle, process and reuse bio-waste; (e) market studies; and (f) political will [83, 84]. It is estimated that education of people at all levels is essential to promote public awareness and minimize environmental pollution. Therefore, to enhance sorting and collection and implement waste management programs, local authorities should identify objectives and constraints, enhance data collection, analyze the principal options and decide the best practices.

On global scale, different separate collection systems and sorting rates have been estimated. Separate collection is part of the comprehensive urban waste management system, which starts with collection and gets to disposal [85, 86]. Technically, once waste have been separated, they are usually stored at home or collected on points located on the streets, according to different techniques: (a) door to door system; (b) kerbside system; (c) drop-off points system; (d) deposit and establishment level; (e) deposit at facility level.

Latest legislative addresses have proposed the harmonization of waste separate collection across Europe [87], as to increase collection rates in already virtuous countries, strengthen sorting capacities of poorly performing regions and tackle local disparities [88, 89]. Such a proposal is based on the assumption that efficient separate collection is a precondition for high-quality recycling and preparation for reuse, preventing hazardous waste from contaminating other waste streams, communities and the environment. Although it is difficult to establish the best system, State members should work together towards the identification of common quality management systems. Further, it has been estimated that door-to-door collection schemes deliver better results and are more likely to involve consumers in the sustainable process [90], making them more aware of the waste they produce [91]. Further, door-to-door collection could be easily monitored, and unsustainable consumers are more likely to be subject to fines [92].

On a European scale, the less virtuous countries are still Malta, Greece and Romania, which account for over 90%, 85% and 80% of landfill rates. Sweden, Germany and Belgium have reached near-zero landfilling, representing at current the best European benchmarks [93]. Italy still presents remarkable room for improvement in landfilling diversion.

## Conclusions

The Italian poultry sector represents an interesting example in the field of meat industries, being the most efficient in terms of yield, compared with beef and pork production. It illustrates an interesting example as regards fresh meat final consumption, either at households or food services, as chicken leg portions, whole chickens, chicken wings and thighs are generally sold and consumed with bones. Nevertheless, one of the main challenges affecting the poultry sector regards the separate collection of its related bio-waste streams. It emerges that more than 144,000 t of bio-waste are still addressed to landfill as unsorted waste, representing at the same time an economic loss and an environmental burden. Considering the theoretical methane potential embedded within sorted poultry waste, it is possible to estimate an amount of more than 104–100 million m^3^. Further, such an amount could be increased by 75–79 million m^3^, if additional chicken bones and skins are properly collected instead of being landfilled.

The research outlines the urgency to increase knowledge on the poultry bio-waste potential among final consumers, stressing the importance of creating high-added value products and bio-methane. It seems that consumers are still too much *unfamiliar* with food waste consequences and bio-waste separate collection, and several efforts to educate consumers to sustainable consumption, food waste prevention or food donation are required. As regards the opportunities offered by the application of the MFA, its application allows at the same time food waste measurement—required by national and international authorities—and the valuation of production chain dysfunctions, both from a technological and a managerial perspective.

As far as future research, the authors intend to explore the correlations and the cause–effect relationships between food choices based on the abandonment of traditional meat or on the adoption of meat substitutes and the amount of bio-waste at country level. It seems appealing to comprehend if bio-waste reduction could be reached by the adoption of new diets and the reduction in traditional meat consumption.

## References

[1] Ismea (2020a) Avicoli e uova e scheda di settore. http://www.ismeamercati.it/flex/ cm/pages/ServeBLOB.php/L/IT/IDPagina/3517 (accessed 18 June 2020)

[2] Statista (2021a) Total number of poultry slaughtered annually in Europe in 2020, by country (in 1,000 heads). Eurostat

[3] Statista (2020) Largest European companies in poultry meat production as of October 2019 (in millions of animals slaughtered annually. WATT Global Media, Illinois, United States

[4] United Nations (2015) Transforming our world: the 2030 Agenda for sustainable development. https://sdgs.un.org/2030agenda (accessed 18 June 2020)

[5] van de Pas B (2020) Meat trends in Europe. Statista DossierPlus on meat trends in Europe. Statista

[6] WWF, Almond REA, Grooten M, Petersen T (2020). Bending the curve of biodiversity loss. WWF.

[7] Amicarelli V, Rana LR, Lombardi M, Bux C (2021). Material flow analysis and sustainability of the Italian meat industry. J Clean Prod.

[8] Yu Q, Li H (2021). Life cycle environmental performance of two restaurant food waste management strategies at Shenzhen, China. J Mater Cycles Waste Manag.

[9] De Mendonca TR, Zhou Y (2019). Environmental performance, customer satisfaction, and profitability: a study among large U.S. companies. Sustainability.

[10] Zylbersztajn D (2017). Agribusiness systems analysis: origin, evolution and research perspectives. Revista de Administração.

[11] CBD (Secretariat of the Convention on Biological Diversity) (2014). Global Biodiversity Outlook 4. Montréal, Canada.

[12] ELD Initiative (2015) The value of land: Prosperous lands and positive rewards through sustainable land management. The Economics of Land Degradation (ELD) Initiative, Bonn, Germany

[13] GSDR (Independent Group of Scientists appointed by the Secretary-General Global Sustainable Development Report) (2019) The future is now – Science for achieving sustainable development. United Nations (UN), New York

[14] FAO (2013) Food wastage footprint. Impacts of natural resources, Summary Report. FAO, Rome

[15] Poore J, Nemecek T (2018). Reducing food’s environmental impacts through producers and consumers. Science.

[16] Our World in Data (2020) Environmental impacts of food production. https://ourworldindata.org/environmental-impacts-of-food#citation (accessed 18 June 2021).

[17] OJEU (Official Journal of European Union) (2009) Regulation (EC) No 1069/2009 of the European Parliament and of the Council of 21 October 2009-laying Down Health Rules as Regards Animal By-Products as Derived Products Not No Hu- man Consumption and Repealing Regulation (EU) No 1774/2002 (Animal By- Products Regulation)

[18] Aan Den Toorn SI, Worrell E, van den Broek MA (2020). Meat, dairy, and more: Analysis of material, energy, and greenhouse gas flows of the meat and dairy supply chains in the EU28 for 2016. J Ind Ecol.

[19] Wyngaard SR, Kissinger M (2019). Materials flow analysis of a desert food production system: the case of bell peppers. J Clean Prod.

[20] Beretta C, Stoessel F, Baier U, Hellweg S (2013). Quantifying food losses and the potential for reduction in Switzerland. Waste Manag.

[21] Padeyanda Y, Jang YC, Ko Y, Yi S (2016). Evaluation of environmental impacts of food waste management by material flow analysis (MFA) and life cycle assessment (LCA). J Mater Cycles Waste Manag.

[22] Cascarosa E, Gea G, Arauzo J (2012). Thermochemical processing of meat and bone meal: a review. Renew Sustain Energy Rev.

[23] Saeid A, Labuda M, Chojnacka K, Gòrecki H (2014). Valorization of bones to liquid phosphorus fertilizer by microbial solubilization. Waste Biomass Valor.

[24] Mirabella N, Castellani V, Sala S (2014). Current options for the valorization of food manufacturing waste: a review. J Clean Prod.

[25] FAO and OECD (2018) Agricultural Outlook 2018–2027. FAO and OECD, Rome, Italy.

[26] Leclercq C, Arcella D, Piccinelli R, Sette S, Le Donne C (2009). The Italian national food consumption survey INRAN-SCAI 2005–06: Main results in terms of food consumption. Public Health Nutr.

[27] Zendehrouhkermani F (2020) Industry revenue of “production of meat and poultry meat products“ in Italy from 2012 to 2024. Statista

[28] Slaboch J, Kotyza P (2016). Comparison of self-sufficiency of selected types of meat in the Visegrad countries. J Central Eur Agric.

[29] Ismea (2020b). Tendenze. Avicoli. https://www.unaitalia.com/wp-content/uploads/2020/07/Report-Ismea-Tendenze-Avicoli-Panoramica-sullannata-2019.pdf (accessed 18 June 2021).

[30] Skunca D, Tomasevic I, Nastasijevic I, Tomovic V, Djekic I (2018). Life cycle assessment of the chicken meat chain. J Clean Prod.

[31] MacLeod M (2013). Nutrition-related opportunities and challenges of alternative poultry production systems. Lohman Inf.

[32] Thushari I, Vicheanteab J, Janjaroen D (2020). Material flow analysis and life cycle assessment of solid waste management in urban green areas. Thailand Sustain Environ Res.

[33] Redzwan G, Amin MM, Zulkarnain NN, Manson MRA, Annuar MSM, Ilham Z (2017). Extrication of biodiesel feedstock from early stage of food waste liquefaction. J Mater Cycles Waste Manag.

[34] NeskovicMarkic D, StevanovicCarapina H, Bjelic D, StojanovicBjelic L, Ilic P, SobotPesic Z, Kikanovic O (2019). Using material flow analysis for waste management planning. Polish J Environ Stud.

[35] Aydin N (2020). Materials flows analysis as a tool to improve solid waste management: A case of Ankara. J Nat Haz Environ.

[36] Cesari V, Zucali M, Sandrucci A, Tamburini A, Bava L, Toschi I (2017). Environmental impact assessment of an Italian vertically integrated broiler system through a life cycle approach. J Clean Prod.

[37] Pishgar-Komleh SH, Akram A, Keyhani A, van Zelm R (2017). Life cycle energy use, costs, and greenhouse gas emission of broiler farms in different production systems in Iran—a case study of Alborz province. Environ Sci Pollut Res.

[38] Restaino GR (1950). The poultry Industry in Southern Italy. World’s Poultry Sci J.

[39] CartoniMacinelli A, Franzoni A, Dal Bosco A, Schiavone A, Mannelli F, Marzoni M, Castellini C (2020). Distribution and consistency of Ancona and Livorno Poultry breed in Central Italy. Ital J Anim Sci.

[40] OJEU (Official Journal of the European Union) (2019) Commission Delegated Decision (EU) 2019/1597 of 3 May 2019 supplementing Directive 2008/98/EC of the European Parliament and of the Council as regards a common methodology and minimum quality requirements for the uniform measurement of levels of food waste, L.248/77. 2019.

[41] Caldeira C, De Laurentiis V, Sala S (2020) Quantification of food waste in EU Member States using material flow analysis. European Union, 2020.

[42] European Commission (2008) Directive 2008/98/EC of the European Parliament and of the Council of 19 November 2008 on waste and repealing certain Directives.

[43] Brunner PH, Rechberger H (2017) Handbook of material flow analysis. for environmental, resource and waste engineers, 2nd edn. CRC Press, Boca Raton

[44] Lederer J, Gassner A, Fellner J, Mollay U, Schremmer C (2021). Raw materials consumption and demolition waste generation of the urban building sector 2016–2050: A scenario-based material flow analysis of Vienna. J Clean Prod.

[45] López de Munain D, Castelo B, Ruggerio CA (2021). Social metabolism and material flow analysis applied to waste management: A study case of Autonomous City of Buenos Aires, Argentina. Waste Manage.

[46] Westbroek CD, Bitting J, Craglia M, Azevedo JMC, Cullen JM (2021). Global material flow analysis of glass. from raw materials to end of life. J Ind Ecol.

[47] Caldeira C, De Laurentiis V, Corrado S, van Holsteijn F, Sala S (2019). Quantification of food waste per product group along the food supply chain in the European Union: a mass flow analysis. Resour Conserv Recycl.

[48] Hendriks CR, Obernosterer D, Müller S, Kytzia P, Brunner BPH (2000). Material flow analysis: a tool to support environmental policy decision making Case-studies on the city of Vienna and the Swiss lowlands. Int J Just Sustain.

[49] Ispra (2019). Rapporto rifiuti urbani. Edizione 2019. Italy, Rome.

[50] Istat (2021). Macellazioni – Carni bianche – Dati annuali. http://dati.istat.it/Index.aspx?DataSetCode=DCSP_MACELLAZIONI (accessed 21 June 2021)

[51] Heinz G, Hautzinger P (2007) Meat processing technology for small to medium scale producers. RAP Publication, 2007/20, FAO, Bangkok.

[52] Hamerschlag K, Venkat K (2011). Meat eater’s guide. To Climate Change þ Health. Lifecycle assessments: methodology and results. Environmental Working Group and Clean Metrics Corp.

[53] Roma R, Corrado S, De Boni A, Bonaventura Forleo M, Fantin V, Moretti M, Palmieri N, Vitali A, De Camillis C (2015) Life cycle assessment in the livestock and derived edible products sector. In: Notarnicola A, et al (eds), Life cycle assessment in the agri-food sector. Springer, Berlin, pp. 251–332

[54] Bekessy SA, Selinske MJ (2017) Chapter 9—Social-ecological analyses for better water resources decisions. In: BT Hart, J Doolan (eds) Decision making in water resources, policy and management. Academic Press, New York, 151–164.

[55] Balaman SY, Balaman SY (2019). Chapter 5—uncertainty issues in biomass-based production chains. Decision-making for biomass-based production chains.

[56] Hayse PL, Marion WW (1973). Eviscerated yield, component parts, and meat, skin and bone ratios in the chicken broiler. Poult Sci.

[57] Seidavi AR, Zaker-Esteghamati H, Scanes CG (2019). Chicken processing: impact, co-products and potential. World’s Poultry Sci J.

[58] Eisenhardt KM (1989). Building theories from case study research. Acad Manag Rev.

[59] Eisenhardt KM, Huberman AM, Miles MB (2002). Building theories from case study research. The qualitative researchers’ companion.

[60] Stake R (2006). Multiple case study analysis.

[61] Ju M, Osako M, Harashina S (2017). Food loss rate in food supply chain using material flow analysis. Waste Manage.

[62] Lombardi M, Rana R, Fellner J (2021). Material flow analysis and sustainability of the Italian plastic packaging management. J Clean Prod.

[63] Nielsen SS, Alvarez J, Bicout DJ, Calistri P, Depner K, Drewe JA, Garin-Bastuji B, Gonzales Rojas JL, Gortázar Schmidt C, Miranda Chueca MÁ, Roberts HC, Sihvonen LH, Spoolder H, Stahl K, Velarde Calvo A, Viltrop A, Winckler C, Candiani D, Fabris C, Van der Stede Y, Michel V, EFSA Panel on Animal Health and Animal Welfare (2019). Scientific opinion on Slaughter of animals: poultry. EFSA J.

[64] Statista (2021b) Resident population of Italy in 2020, by region. Statista Research Department.

[65] Garcia-Garcia G, Stone J, Rahimifard S (2019). Opportunities for waste valorisation in the food industry—A case study with four UK food manufacturers. J Clean Prod.

[66] Nayak A, Bhushan B (2019). An overview of the recent trends on the waste valorization techniques for food wastes. J Environ Manage.

[67] Galanakis, C. (2020). Food waste valorization opportunities for different food industries. In: The Interaction of Food Industry and Environment. Ed.: Charis Galanakis. Academic Press, 341–422.

[68] Rajeh C, Saoud IP, Kharroubi S, Naalbandian S, Abiad MG (2021). Food loss and food waste recovery as animal feed: a systematic review. J Mater Cycles Waste Manage.

[69] WRAP (2021) WRAP and the Circular Economy. https://wrap.org.uk/about-us/our-vision/wrap-and-circular-economy (accessed on 30 June 2021).

[70] Raggi A, Petti L, De Camillis C, Mercuri L, Pagliuca G, Puig R (2007). Cattle slaughtering residues: Current scenario and potential options for slaughterhouses in Abruzzo. Industrial Ecology in the Cattle-to-Leather Supply Chain.

[71] Galanakis, C. (2021). Food waste valorization opportunities for different food industries. In: The Interaction of Food Industry and Environment, Ed. Galanakis, C. Elsevier Inc. Academic Press.

[72] Tricase C, Lombardi M (2009). State of the art and prospects of Italian biogas production from animal sewage: technical-economic considerations. Renew Energy.

[73] Boles J, Rathgeber B, Shand P (2000). Recovery of proteins from beef bone and the functionality of these proteins in sausage batters. Meat Sci.

[74] Barakat NAM, Khil MS, Omran AM, Sheikh FA, Kim HY (2009). Extraction of pure natural hydroxyapatite from the bovine bones bio waste by three different methods. J Mater Process Technol.

[75] Kew SJ, Gwynne JH, Enea D, Abu-Rub M, Pandit A, Zeugolis D, Brooks RA, Rushton N, Best SM, Cameron RE (2011). Regeneration and repair of tendon and ligament tissue using collagen fibre biomaterials. Acta Biomater.

[76] Lee SJ, Kim YS, Hwang JW, Kim EK, Moon SH, Jeon BT, Jeon YJ, Kim JM, Park PJ (2012). Purification and characterization of a novel antioxidative peptide from duck skin by-products that protects liver against oxidative damage. Food Res Int.

[77] Wang S, Jena U, Das KC (2018). Biomethane production potential of slaughterhouse waste in the United State. En Conv Manage.

[78] Agovino M, Garofalo A, Mariani A (2017). Separate waste collection in Italy: the role of socio-cultural factors and targets set by law. Environ Dev Sustain.

[79] Saladié O, Santos-Lacueva R (2016). The role of awareness campaigns in the improvement of separate collection rates of municipal waste among university students: a causal chain approach. Waste Manage.

[80] Xu L, Ling M, Wu Y (2018). Economic incentive and social influence to overcome household waste separation dilemma: a field intervention study. Waste Manage.

[81] Bryant CJ (2020). Culture, meat, and cultured meat. J Anim Sci.

[82] Chriki S, Hocquette JF (2020). The myth of cultured meat: a review. Front Nutr.

[83] Polprasert C (2007). Organic waste recycling. Technology and management.

[84] Polprasert C, Koottatep T (2017) Organic waste recycling: technology, management and sustainability. IWA Publishing, London, pp 1–576.

[85] Gallardo A, Prades M, Bovea MD, Colomer FJ (2011). Separate Collection Systems for Urban Waste (UW). Manag Organ Waste.

[86] Gallardo A, Colomer-Mendoza FJ, Carlos-Alberola M, Badenes C, Edo-Alcòn N, Esteban-Altabella J (2021). Efficiency of a pilot scheme for the separate collection of the biowaste from municipal solid waste in Spain. Sci Rep.

[87] European Environmental Bureau and Zero Waste Europe (2020). Harmonisation of waste separate collection. https://zerowasteeurope.eu/wp-content/uploads/2020/07/2020_07_14_zwe_eeb_position-paper_harmonisation-of-waste-separate-collection_en.pdf (accessed on 26 November 2021).

[88] Bergeron FC (2018). Waste management assessment in Geneva through material system and resource analysis. J Mater Cycles Waste Manage.

[89] Mihai FC (2018). Waste collection in rural communities: challenges under EU regulations. A case study of Neamt County, Romania. J Mater Cycles Waste Manage.

[90] Kormaňáková M, Remešová M, Vančová T. Food waste in municipal mixed waste produced at household level: empirical evidence from the Czech Republic. Journal of Material Cycles and Waste Management 23:1348–1364.

[91] Jigani AI, Delcea C, Ioanăș C (2020). Consumers’ behavior in selective waste collection: a case study regarding the determinants from Romania. Sustainability.

[92] Laurieri N, Lucchese A, Marino A, Digiesi S (2020). A door-to-door waste collection system case study: a survey on its sustainability and effectiveness. Sustainability.

[93] European Environmental Agency (2021). Diversion of waste from landfill in Europe. https://www.eea.europa.eu/ims/diversion-of-waste-from-landfill (accessed on 26 November 2021).

